# Risk Minimization of Hemolytic Disease of the Fetus and Newborn Using Droplet Digital PCR Method for Accurate Fetal Genotype Assessment of *RHD*, *KEL*, and *RHCE* from Cell-Free Fetal DNA of Maternal Plasma

**DOI:** 10.3390/diagnostics11050803

**Published:** 2021-04-28

**Authors:** Radek Vodicka, Jana Bohmova, Iva Holuskova, Eva Krejcirikova, Martin Prochazka, Radek Vrtel

**Affiliations:** 1 Department of Medical Genetics, University Hospital and Palacky University Olomouc, 775 20 Olomouc, Czech Republic; vodickar@fnol.cz (R.V.); eva.krejcirikova@fnol.cz (E.K.); martin.prochazka@fnol.cz (M.P.); radek.vrtel@fnol.cz (R.V.); 2 Department of Blood Transfusion, University Hospital and Palacky University Olomouc, 775 20 Olomouc, Czech Republic; iva.holuskova@fnol.cz

**Keywords:** cell-free fetal DNA, hemolytic disease of fetus and newborn, *RHD*, *RHCE*, *KEL*, droplet digital PCR, noninvasive fetal genotyping, blood group incompatibility

## Abstract

The molecular pathology of hemolytic disease of the fetus and newborn (HDFN) is determined by different *RHD*, *RHCE*, and *KEL* genotypes and by blood group incompatibility between the mother and fetus that is caused by erythrocyte antigen presence/absence on the cell surface. In the Czech Republic, clinically significant antierythrocyte alloantibodies include anti-D, anti-K, anti C/c, and anti-E. Deletion of the *RHD* gene and then three single nucleotide polymorphisms in the *RHCE* and *KEL* genes (rs676785, rs609320, and rs8176058) are the most common. The aim of this study is to develop effective and precise monitoring of fetal genotypes from maternal plasma of these polymorphisms using droplet digital (dd)PCR. Fifty-three plasma DNA samples (from 10 to 18 weeks of gestation) were analyzed (10 *RHD*, 33 *RHCE*, and 10 *KEL*). The ddPCR methodology was validated on the basis of the already elaborated and established method of minisequencing and real-time PCR and with newborn phenotype confirmation. The results of ddPCR were in 100% agreement with minisequencing and real-time PCR and also with newborn phenotype. ddPCR can fully replace the reliable but more time-consuming method of minisequencing and real-time PCR *RHD* examination. Accurate and rapid noninvasive fetal genotyping minimizes the possibility of HDFN developing.

## 1. Introduction

Hemolytic disease of the fetus and newborn (HDFN) is a disease that can cause perinatal morbidity and mortality [1,2,3,4]. Cellular and molecular pathogenesis of HDFN lies in maternal erythrocyte alloimmunization, which develops as a result of stimulation of the immune system by “foreign” antigens on the surface of fetal red blood cells. Maternal IgG alloantibodies are formed and cross the placenta, bind to fetal erythroid cells with unknown antigens, and then are destroyed in the reticuloendothelial system in the fetal spleen. Immune hemolysis can then cause varying degrees of fetal anemia. In the most severe cases, the fetus may die of heart failure in utero. In neonates, it can lead to severe forms of neonatal hyperbilirubinemia at risk of kernicterus [5,6,7,8,9,10]. Erythrocyte alloimmunization can be induced by blood transfusion containing a foreign erythrocyte antigen or during pregnancy as a result of feto-maternal hemorrhage, if the fetus has inherited an antigen from the father that is not present on the mother’s erythrocytes. The first contact of the maternal immune system with an incompatible fetal erythrocyte antigen usually does not produce a severe form of HDFN. Antigen-incompatible fetuses are usually at risk of HDFN only in subsequent pregnancies [11,12,13]. Unlike alloantibodies directed against Rh antigens, anti-K antibodies cause suppression of erythropoiesis rather than hemolysis of incompatible erythrocytes. The K antigen is expressed by erythroid precursor cells, in contrast to Rh proteins, which are expressed on the surface of mature erythrocytes. In this case, the level of maternal anti-K antibodies is not a good indicator of HDFN and the severity of HDFN is difficult to predict because the correlation between alloantibody level and degree of fetal anemia is very small [14,15].

*RHD, RHCE*, and *KEL* blood incompatibilities between the mother and fetus are among the most clinically serious in the Czech Republic in terms of possible development of HDFN. Clinically significant anti-erythrocyte alloantibodies include anti-D, anti-K/k, anti C/c, and anti-E/e. The presence or absence of an erythrocyte antigen on the cell surface is usually directly related to a particular genotype. In the European population, it is most often a complete deletion of the *RHD* gene and three single nucleotide polymorphisms (SNP) in the *RHCE* and *KEL* genes (rs676785, rs609320, and rs8176058).

Rh system antigens are encoded by two genes, *RHD* and *RHCE*. Both the genes are composed of 10 coding exons and have 94% sequence homology, which is evolutionarily conditioned by tandem duplication [16]. The most immunogenic and clinically important antigen of the Rh system is the D antigen [17]. The *RHD* negative genotype in the White population and thus the absence of the D antigen in the erythrocyte membrane is due to the deletion of the *RHD* gene in the homozygous state [16,18]. About 15–17% of the White population has the *RHD* negative genotype [17].

The E/e antigen differs based on an SNP (rs609320, c.676G > C) in exon 5, which results in a Pro226Ala substitution in the fourth extracellular loop of the transmembrane protein [19]. The frequency of the E antigen in the White population reaches about 29%; the frequency of the e antigen is 98% [20]. C/c antigens differ by six nucleotides, which encode four amino acid substitutions: Cys16Trp, He60Leu, Ser68Asn, and Ser103Pro. The C/c antigen specificity is determined by only one SNP (rs676785, c.307G > A), which results in a Ser103Pro substitution [19]. The frequency of the C antigen in the White population reaches about 68%; the frequency of the c antigen is 80% [20].

In addition to alloimmunization in the Rh system, Kell alloimmunization is another common cause of hemolytic disease of the fetus and newborn in the White population. The *KEL* gene has two major codominant alleles: *KEL1* and *KEL2*. The difference in the gene sequence between the *KEL1* and *KEL2* alleles is caused by a single nucleotide substitution in exon 6 (rs8176058, c.578C > T). The *KEL1* allele is given by the presence of the T nucleotide and encodes the K antigen, the presence of the C nucleotide characterizes the *KEL2* allele and encodes the K antigen [21,22]. The single nucleotide substitution 578C > T results in the Thr193Met substitution [23,24]. The K antigen is present in 9% of the White population [25].

The aim of this work was to utilize the ddPCR method for noninvasive genotyping of the *RHD* allele, the *KEL1* allele of the *KEL* gene, and the C or c and E alleles of the *RHCE* gene to design a reliable diagnostic strategy that is applicable to routine practice.

## 2. Materials and Methods

### 2.1. Sample Collection

Plasma samples and control plasma DNA for ddPCR assay specificity assessment were collected in collaboration with the Department of Medical Genetics, the Department of Obstetrics and Gynecology, and the Department of Transfusion Medicine of the University Hospital Olomouc. All of the subjects enrolled in the study signed an informed consent form approved by the Ethics Committee of the University Hospital Olomouc (approval code: 150/10). The samples that had already been tested by validated TaqMan real-time PCR and minisequencing methods [26,27,28] were used to validate and assess the ddPCR methodology. The correlation between fetal genotype and phenotype was further verified in the newborn using an immunoassay. The characterization of the tested plasma sample group for particular blood groups is in Table 1. Two positive and two negative control plasma DNA samples were used for each tested ddPCR assay. In these control samples, cffDNA genotypes were verified using DNA from neonatal buccal swabs.

### 2.2. Sample Preparation and DNA Isolation

All 53 plasma samples and 8 control samples from pregnant subjects were collected into 9-mL tubes containing ethylenediaminetetraacetic acid (EDTA). Anticoagulated blood was placed on ice immediately after collection and was processed up to 4 h after sampling. Plasma was separated from the cellular fraction of blood using double centrifugation at 4 °C (1600× *g* for 10 min and 16,000× *g* for 10 min). The plasma samples were frozen until further processing at −80 °C. Plasma-cell-free (cf) DNA was isolated from 5 mL of plasma using the QIAamp Circulating Nucleic Acid Kit (Qiagen, Venlo, The Netherlands).

### 2.3. Determination of Fetal Blood Genotype by ddPCR

Two assays were designed for each tested blood group. The ddPCR assays tested for each genotype were designed to keep the length of the PCR products as short as possible. Specificity of primers and probes were optimized using control samples by two annealing temperatures. The sequence of β-globin was used as an internal control to amplify and quantify the ddPCR RHD assay. Conditions of testing and optimization of ddPCR assays are stated in Table 2.

The reactions mix for DNA isolated from the plasma were prepared in a 22-µL volume. Premix contained 11 µL of 2× ddPCR Supermix for Probes (no dUTP) (Bio Rad, Hercules, CA, USA), 1.1 µL 20× target primers/probe (FAM) in the final concentration of primer 9 pmol/L^−1^ and probe 2.5 pmol/L^−1^) (Integrated DNA Technologies, Inc. (IDT),Coralville, Iowa, USA), 1.1 µL 20× target primers/probe (HEX) in the final concentration of primer 9 pmol/L^−1^ and probe 2.5 pmol/L^−1^) (IDT), and 8.8 µL plasma DNA or PCR water (Top-Bio, Prague, Czech Republic) as NTC. Reaction mix for commercial ddPCR mutation assay:KEL p.T193M, human was prepared in a 22 µL volume. Premix contained 11 µL of 2x ddPCR supermix for probes (no dUTP) (Bio Rad), 1.1 µL assay (FAM + HEX), 1.1 µL PCR water, and 8.8 µL plasma DNA or PCR water (Top-Bio) as NTC. The droplets were generated from reaction mixes and droplet generation oil for probes (Bio Rad) in the QX200 Automated Droplet Generator in a final volume of 40 µL. After droplet generation, thermal cycling proceeded in the C100 Touch Thermal Cycler (Bio Rad). Amplification was performed by ramp rate 2 °C/s under the following conditions: 95 °C 10 min, 40 PCR cycles with 94 °C 30 s, 55 °C, or 60 °C 60 s profile, 98 °C 10 min. After thermal cycling, samples were placed in the QX200 Droplet Reader (Bio Rad). The QuantaSoft Software version 1.7.4 (Bio Rad) was used for assay analysis and evaluation.

## 3. Results

Basic ddPCR parameters and cell-free plasma DNA (cffDNA) genotype results are shown in Table 3. All the results were in 100% agreement with the minisequencing and real-time PCR and with the newborn phenotype.

## 4. Discussion

The incompatibility of the RhD system is considered to be the most clinically significant. Most laboratories that provide noninvasive cffDNA *RHD* genotyping perform a real-time PCR test for the presence or deletion of the *RHD* gene. Our previous study confirmed that for the Czech population RhD negativity is almost exclusively associated with the deletion of the entire *RHD* gene [26].

Other variants associated with blood incompatibility may also have clinical impacts in the Czech and/or in Central European population, especially *RHCE* and *KEL* genes. For *RHCE* and *KEL*, it is necessary to distinguish an SNP between the homozygous mother and heterozygous fetus.

RHD tests using real-time PCR and RHCE and KEL using minisequencing have already been introduced at our workplace [26,27,28]. One of the aims of the study was to methodically unify both procedures and, in the case of *RHCE* and *KEL*, to simplify and speed up the examination.

To optimize the ddPCR assays, primers and probes were designed (Table 2) to meet the following criteria: the primer and probe sequence specificity (neither the primers nor the probes should interfere in other than desired polymorphic sites), GC percentage, similar primer and probe Tm, and the length of the PCR product (ideally below 100 bp).

The assays that showed minimum (ideally zero) non-specific positive droplets and the assays with the shortest PCR products were selected for the subsequent validation and confirmation study.

**Figure 1:**
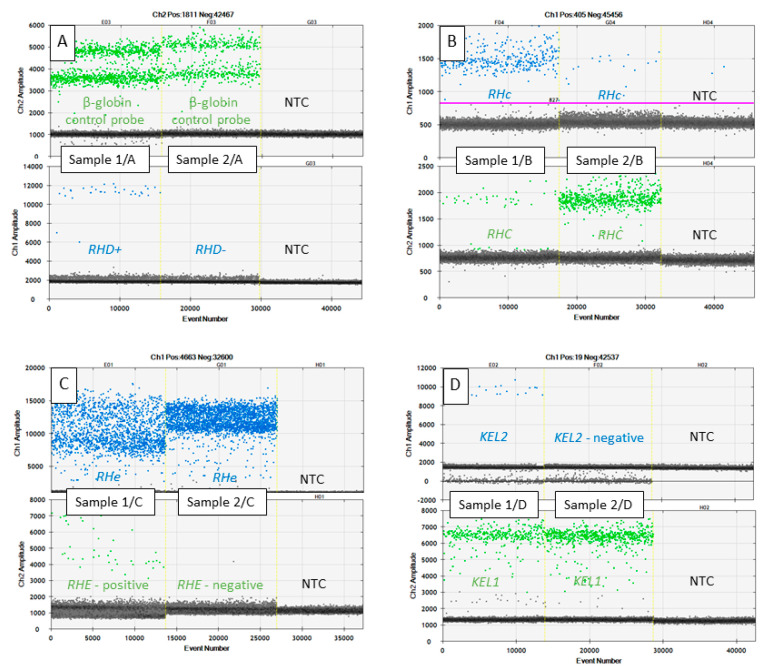
Graphical outputs from one dimensional graph between the fluorescence intensity (*y*-axis) and droplet number (*x*-axis) of *RHD*, *RHCE*, and *KEL* genotyping from maternal cell-free plasma DNA; (**A**) green channel indicates plasma DNA control β-globin amplification, blue channel indicates *RHD* exon 7 positive droplets originate from the fetus. Maternal genotype in Sample 1/A is *RHD−/RHD−* while the fetal genotype is *RHD+/RHD−*, maternal genotype is *RHD−/RHD−* and the fetal genotype is also *RHD−/RHD−* in Sample 2/A; (**B**) blue channel positive droplets indicate variant *RHc* and green channel indicates *RHC* variants, maternal genotype in Sample 1/B is *RHcc* while fetal genotype is *RHCc*, maternal genotype is *RHCC* and fetal genotype is *RHCc* in Sample 2/B. In the case of RHc measurements, the QuantaSoft software was not able to set the cut-off line so it had to be done manually. Therefore, the purple cutoff line is visible; (**C**) green channel positive droplets indicate variant *RHE*, blue channel positive droplets indicate variant *RHe*. Plasma DNA sample 1/C contains both the maternal *RHee* genotype and different *RHEe* fetal genotype. The same genotypes *RHee* (maternal and fetal) are shown in the sample 2/C; (**D**) blue channel positive droplets indicate *KEL1* variant, green channel positive droplets indicate *KEL2* variant. Plasma DNA sample 1/D contains both the maternal *KEL2/KEL2* genotype and different *KEL1/KEL2* fetal genotype. Sample 2/D illustrates the same *KEL2/KEL2* genotypes. NTC: Non-template control.

The basic characteristics of ddPCR were assessed for individual assays and the percentage of fetal fraction was estimated (Table 3, Figure 1). The total number of droplets ranged from 11,000 to 18,000. The vast majority were negative. For total plasma DNA, the number of positive droplets ranged from 376 to 2875. When only the fetal DNA fraction were assessed, the number of positive droplets ranged from 8 to 91.

Due to the low percentage of positive droplets, the calculation of the amount of fetal fraction was based on the assumption that one DNA molecule occupies one droplet. Another assumption is that the maternal genotype is in a homozygous status and if the fetal genotype is different from the maternal one, it must therefore be in a heterozygous status.

The percentage of fetal fraction is very variable from 3.5% to 26%, which corresponds to other studies [29,30,31,32,33]. We did not find any correlation when assessing the total and fetal fraction.

Comparison of real-time PCR and ddPCR methodology in noninvasive *RHD* fetal genotyping:

ddPCR accuracy according to our results is comparable with real-time PCR; this is in agreement with the study by Svobodova et al., 2015 [34]. Sillence et al., 2015 demonstrated, on the basis of the sequence from the *SRY* gene, higher sensitivity of ddPCR with comparison to real-time PCR [35].

Analysis duration and economic aspects are very similar. In addition, by ddPCR it is possible to absolutely quantify the amount of cffDNA.

Comparison of minisequencing with ddPCR methodology:

Noninvasive genotyping of SNPs using real-time PCR may be limited due to the non-specific fluorescent background [27]. To avoid the real-time PCR limitation, the minisequencing methodology was optimized and validated. The minisequencing method is able to determine fetus specific SNP if the amount of cffDNA fraction is as low as 1% [27]. Compared to ddPCR, the minisequencing methodology is more laborious. Briefly, after the 1st PCR, purification follows, and the next step is the minisequencing PCR, followed by further purification and separation by capillary electrophoresis. Due to the many steps and procedures, the whole test takes 2 days to complete; ddPCR can be performed in half a working day. Based on our study, the accuracy test is comparable; moreover, similarly to *RHD* genotyping, the amount of cffDNA can be exactly measured and quantify.

Due to the very “clean” NTC histogram profile, it is possible to empirically set the limit of the maximal number of false positive droplets to 4 [36]. The base of our study resulted in the minimal limit of fetal fraction positive droplets that can be estimated to the limit value of 5. The total amount of cffDNA according to the ddPCR results of samples with different genotypes was sufficiently robust so the probability of false negative results was minimized. Nevertheless, the analysis with the result around the minimal positive droplet limit should always be repeated.

Few studies have been performed regarding the similar genotype spectrum of *RHD, KEL*, and *RHCE* blood incompatibility. Finning et al. (2007) successfully tested the real-time PCR method to test the same variant spectrum as in our study [37]. Cro et al. (2016) described noninvasive Real-time PCR *KEL* genotyping by incorporating allele-specific probes [38]. O’Brien et al. (2020) published a study that describes ddPCR utilization for noninvasive testing of fetal variants associated with Kell, RhCE, and Duffy antigens [39].

The NGS (next generation sequencing) methodology can also be used to accurately determine fetal blood group genotyping [40].

ddPCR is widely used for noninvasive prenatal detection of various types of mutations in many genetic diseases such as monogenic hereditary diabetes mellitus [41] and mutations in the *NF1* [42] and *CFTR* genes [42,43].

The Czech population spectrum of investigated polymorphisms that may lead to HDFN was set on the basis of a wide discussion of leading experts in the Czech Republic addressing the monitoring of pregnancies at risk of HDFN.

The open ddPCR platform offers very flexible potential extensions with additional blood incompatibility assays if required.

## 5. Conclusions

ddPCR is a generally useful tool for detecting cffDNA from maternal plasma. We adapted the method for the genotyping and assessment of fetal genetic variants that are responsible for the clinically most significant antierythrocyte alloantibodies, anti-D, anti-K, anti C/c, and anti-E, and may result in HDFN development. The method can fully replace the reliable but time-consuming method of minisequencing, and *RHD* examination using real-time PCR can be replaced by ddPCR. Accurate and rapid fetal genotyping of *RHD, RHCE*, and *KEL* minimizes the risk of HDFN developing.

## Figures and Tables

**Table 1:** Characterization of the sample group.

Pregnant Woman Blood Antigen	*n* = 53	Confirmation Method	Newborn Phenotype	Gestation Week	Gestation Week		Age	Age		BMI	BMI		Ethnic Group of Participants
					Median	Mean		Median	Mean		Median	Mean	
“D-antigen” negative pregnant women (genotype *RHdd*)	10	Real-Time PCR	5 RhD+ 5 RhD−	10–18	12	12.5	18–43	29	30	17–36	23	24.3	Caucasian
“C-antigen” pregnant women (genotype *RHCC*)	11	Minisequencing	4 RhC 7 Rhc										
“c-antigen” pregnant women (genotype *RHcc*)	6	Minisequencing	4 RhC 2 Rhc										
“e-antigen” pregnant women (genotype *RHee*)	16	Minisequencing	12 Rhe 4 RhE										
“k-antigen” pregnant women (genotype *KEL2/KEL2*)	10	Minisequencing	5 Kell− 5 Kell+										

**Table 2:** Conditions of testing and optimization of ddPCR assays.

Gene, SNP rs Number	Allele	Primer F (5′–3′)	Primer R (5′–3′)	Probe (5′–3′)			PCR Product bp	Specificity to cffDNA	Optimal Annealing Temperature	
				Fluorophore	Sequence of the Probe	Quencher			55 °C **	60 °C
*RHD*	*RHD*	GGGTGTTGTAACCGAGTGCTG	CTCCAAGCAGACCCAGCAAG	56-FAM	CCCACAGCTCCATCATGGGCTACAA	3IAkFQ	72	* YES	YES	YES
		GGGTGTTGTAACCGAGTGCTG	CCGGCTCCGACGGTATC				134	YES	YES	YES
*β-globin*	*---*	GTGCATCTGACTCCTGAGGAGA	CCTTGATACCAACCTGCCCAG	5HEX	AAGGTGAACGTGGATGAAGTTGGTGG	3IAkFQ	74	* YES	YES	YES
*RHCE*, rs609320	*RHE*	ATTCTTCCTTTGGATTGGAC	CTTGTGGATGTTCTGGC	5HEX	TCAGCAGAGGAGAGTTG	3IAkFQ	64	* YES	YES	YES
		TGGCATTCTTCCTTTGGATTGG	CTCAGACCTTTGGAGCAGGAGT				134	YES	YES	YES
	*RHe*	ATTCTTCCTTTGGATTGGAC	CTTGTGGATGTTCTGGC	56-FAM	TCAGCAGAGCAGAGTTG	3IAkFQ	64	* YES	YES	YES
		TGGCATTCTTCCTTTGGATTGG	CTCAGACCTTTGGAGCAGGAGT				134	YES	YES	YES
*RHCE*, rs676785	*RHC*	AATACCTGAACAGTGTGATG	CTGCTGGACGGCTTC	5HEX	CCTTCCCAGAAGGGAAC	3IAkFQ	74	* YES	YES	NO
		CCAGCCACCATCCCAATACC	TGTGCAGTGGGCAATCCTG				94	YES	YES	NO
	*RHc*	AATACCTGAACAGTGTGATG	CTGCTGGACGGCTTC	56-FAM	GATGACCACCTTCCCAGGAGGGAA	3IAkFQ	74	* YES	YES	NO
		CCAGCCACCATCCCAATACC	TGTGCAGTGGGCAATCCTG				94	YES	YES	NO
*KEL*, rs8176058	*KEL1*	ddPCR Mutation Assay: KEL p.T193M, Human		56-FAM	ddPCR Mutation Assay: KEL p.T193M, Human	3IAkFQ	65	* YES	YES	NO
		GGTAAATGGACTTCCTTAAAC	CTGAAGAAAGGGAAATGG	56-FAM	TAACCGAATGCTGAGACTTCTGATGAGTCAG	3IAkFQ	77	NO	NO	NO
	*KEL2*	ddPCR Mutation Assay: KEL p.T193M, Human		5HEX	ddPCR Mutation Assay: KEL p.T193M, Human	3IAkFQ	65	* YES	YES	NO
		GGTAAATGGACTTCCTTAAAC	CTGAAGAAAGGGAAATGG	5HEX	TAACCGAACGCTGAGACTTCTGATGAGTCAG	3IAkFQ	77	NO	NO	NO

* Assays selected for ddPCR as proper for plasma samples analysis. ** Annealing temperature 55 °C was used for plasma samples analysis, as optimal.

**Table 3:** Results of *RHD, RHCE*, and *KEL* genotyping from maternal cffDNA and basic ddPCR parameters.

Maternal Genotype (*n*)	Fetal Genotype */Phenotype ** (*n*)	FAM (Blue) Channel Positive Droplets Mean (Min; Max)	VIC (Green) Channel Positive Droplets Mean (Min; Max)	Negative Droplets Mean (Min; Max)	Fetal Fraction Percentage Mean (Min; Max) ***
*RHD−* (10)	*RHD+/*RhD+ (5)	47.6 (28; 91)	1540 (467; 2875)	13,761 (11,583; 16,561)	9% (5.6%; 25%)
	*RHD−/*RhD− (5)	0.6 (0; 2)	1457 (562; 2767)	14,240 (10,471; 17,560)	
*RHee* (16)	*RHee* (12)/Rhee(12)	792 (295; 1809)	0.4 (0; 1)	11,735 (10,417; 13,687)	
	*RhEe* (4)/RhEe (4)	1450 (289; 2854)	25.6 (8; 49)	12,479 (9096; 14,939)	7% (3.5%;11%)
*RHCC* (11)	*RHCc* (4)/RhCc (4)	21 (18; 26)	672 (522; 805)	13,706 (11,718; 17,142)	8% (6.6–10%)
	*RHCC* (7)/RhCC (7)	1 (0;3)	938 (239; 2474)	12,669 (10,528; 13,717)	
*RHcc* (6)	*RHCc* (2)/RhCc (2)	379 and 517	44 and 54		26% and 23%
	*RHcc* (4)/Rhcc(4)	695 (390; 1284)	1.4 (0; 5)		
*KEL2* (10)	*KEL1* (5)/K (5)	22 (17; 31)	760 (376; 1341)	14,237 (13,558; 15,069)	7% (5.5%; 11%)
	*KEL2* (5)/k (5)		625 (440; 922)	14,328 (13,598; 15,321)	
NTC (23)	NTC (23)	0.55 (0; 3)	0.22 (0; 4)	15,646 (12,448; 17,735)	

* Confirmed by minisequencing and by real-time PCR; ** confirmed by newborn phenotype immunotest; *** fetal fraction (cffDNA fraction) was calculated with the assumption that maternal genotype is in homozygous status and that the droplets, if positive, contain only one specific DNA molecule. CffDNA fraction calculation formula: number of the only fetus specific positives (FSP) divided by ((the number of maternal specific positives (MSP) minus FSP) divided by 2)); NTC–non-template control.

## Abbreviations

β-globin	hemoglobin subunit beta
cfDNA	cell free deoxyribonucleic acid
cffDNA	cell free fetal deoxyribonucleic acid
CFTR	cystic fibrosis transmembrane conductance regulator
ddPCR	droplet digital polymerase chain reaction
DNA	deoxyribonucleic acid
dUTP	2´-deoxyuridine, 5´-triphosphate
EDTA	ethylenediaminetetraacetic acid
FSP	fetus specific positives
HDFN	Hemolytic disease of the fetus and newborn
KEL	Kell metallo-endopeptidase
MSP	maternal specific positives
NF1	neurofibromin 1
NGS	next generation sequencing
NTC	non-template control
PCR	polymerase chain reaction
Rh	Rhesus
RHCE	Rh blood group CcEe antigens
RHD	Rh blood group D antigen
SNP	single nucleotide polymorphisms
SRY	sex-determining region
Tm	melting temperature
